# Thorough Wide-Temperature-Range Analysis of Pt/SiC and Cr/SiC Schottky Contact Non-Uniformity

**DOI:** 10.3390/ma17020400

**Published:** 2024-01-13

**Authors:** Razvan Pascu, Gheorghe Pristavu, Dan-Theodor Oneata, Gheorghe Brezeanu, Cosmin Romanitan, Nikolay Djourelov, Andrei Enache, Florin Draghici, Andrei Mario Ivan, Emilian Ceuca

**Affiliations:** 1 National Institute for Research and Development in Microtechnologies—IMT Bucharest, 126A, Erou Iancu Nicolae Street, 077190 Bucharest, Romania; razvan.pascu@imt.ro (R.P.); cosmin.romanitan@imt.ro (C.R.); 2 Faculty of Electronics, Telecommunications and Information Technology, National University of Science and Technology Politehnica Bucharest, 060042 Bucharest, Romania; dan_theodor.oneata@upb.ro (D.-T.O.); andrei.enache1512@upb.ro (A.E.); florin.draghici@upb.ro (F.D.); 3 Extreme Light Infrastructure-Nuclear Physics (ELI-NP), Horia Hulubei National R&D Institute for Physics and Nuclear Engineering (IFIN-HH), 077125 Magurele, Romania; nikolay.djourelov@eli-np.ro; 4 Faculty of Industrial Engineering and Robotics, National University of Science and Technology Politehnica Bucharest, 060042 Bucharest, Romania; andrei_mario.ivan@upb.ro; 5 Department of Informatics, Mathematics and Electronics, Faculty of Exact Sciences and Engineering, University “1 Decembrie 1918” of Alba Iulia, No. 5 Gabriel Bethlen Street, 510009 Alba Iulia, Romania; emilian.ceuca@uab.ro

**Keywords:** Schottky contact, silicon carbide, *p-diode* model, non-uniformity

## Abstract

This paper evaluates the non-uniformity degree of platinum and chromium Schottky contacts on silicon carbide. The forward characteristics of experimental samples were acquired in a wide, 60–500 K, temperature range. Microstructural and conventional electrical characterizations were performed, revealing the presence of inhomogeneities on the contact surface. The main parameters were extracted using inhomogeneity models of varying complexity levels. Their relevance is discussed with respect to the models’ applicable, limited, temperature ranges. Finally, complete forward curve fitting was achieved using *p-diode* modeling, evincing that each type of contact behaves as four parallel-connected ideal diodes. Since these parallel diodes have varying influences on the overall device current with temperature and bias, operable domains can be identified where the samples behave suitably.

## 1. Introduction

Reliable operation in harsh environments and over a wide range of temperatures is a fundamental requirement in many industrial applications [1,2], including cement manufacturing [3], drilling [4], geothermal systems [5], aerospace [6], etc. The working conditions in these applications often include strong vibrations, corrosion, radiation, and elevated heat levels, as well as a significant number of thermal cycles for the involved devices [3].

For such hostile environments, silicon carbide (SiC)-based sensors have emerged as a promising solution [3], especially for applications involving gas concentration measurement [7,8] or temperature sensing [3,9,10,11,12]. Due to the wide bandgap (3.24 eV for 4H-SiC) and low intrinsic carrier concentration, SiC devices can operate at temperatures far above the limits for conventional semiconductors [3]. Furthermore, the mechanical robustness, radiation hardness, chemical inertness, and high thermal conductivity of SiC allow for the operation of these devices under harsh conditions [13].

The simplest semiconductor device that can be fabricated is the Schottky barrier diode (SBD), as the process involves a metal deposition followed by annealing in order to obtain a rectifying contact [14]. Consequently, an SBD is also the most cost-effective and technologically mature device fabricated using SiC [3]. Reported applications range from high-voltage, high-power circuits, such as traction inverters [15], to sensors destined for harsh environmental conditions [3,7].

Over time, different metals have been used for the Schottky contact on SiC substrates, including nickel [3], titanium [16], platinum [17,18] and, less frequently, chromium [19,20]. While Pt is suitable for applications in a wide temperature range, Cr is also an attractive proposition as a Schottky metal because it forms both silicides and carbides with SiC (very stable compounds), resulting in good contact adhesion and mechanical properties [19].

Irrespective of the utilized metal, and even after annealing, contact inhomogeneity still appears, with an observable effect on the Schottky barrier height (SBH) [14,21,22]. Consequently, evaluating the performance of an SBD is still challenging, despite there being more than fifty years of investigations [23]. Therefore, in order to fully understand the behavior of these inhomogeneous contacts, comprehensive characterization needs to be carried out over large temperature spans. The resulting characteristics must be parameterized using specialized models, which need to accurately explain the electrical behavior across the entire range. Modeling accuracy is especially critical for sensing applications, where the SBD model must allow for the precise determination of the sensed quantity (temperature, gas concentration, etc.) based on the measured electrical quantity (voltage or current); thus, the model needs to account for the effects of contact inhomogeneity.

In this paper, the fabrication and electrical characterization of Pt/4H-SiC and Cr/4H-SiC Schottky barrier diodes are presented. The characterization was carried out over a broad temperature domain (60–500 K). The resulting forward curves were comprehensively parameterized using state-of-the-art methods, confirming the presence of contact inhomogeneity.

Afterwards, complete curve fitting was carried out using our recently proposed *p-diode* technique [14,24], which modeled the inhomogeneous SBD as a minimal number of parallel diodes. The resulting excellent fitting accuracy demonstrates that the *p-diode* model can fully explain the forward behavior of the sample over the entire temperature and bias intervals.

## 2. Materials and Methods

In this work, two metals with major differences in their work function (WF) values were used to fabricate Schottky barrier diodes on nitrogen-doped 4H-SiC wafers with an 8 μm epitaxial layer. The SiC substrate was heavily doped, and the epitaxial layer had a doping concentration of around 10^16^ cm^−3^. After the standard RCA chemical cleaning was performed, two different types of SiO_2_ layers were deposited by the low-pressure chemical vapor deposition (LPCVD) method in order to obtain a ramp profile as a termination for the Schottky contact. This technological process is described in detail in refs. [3,25]. The Schottky contact had a circular configuration with a diameter of 400 µm [25], as defined by standard photolithography techniques (lift-off for Pt and wet etching for Cr contacts) in the deposited field oxide (LPCVD). Due to the very high annealing temperature constraint, the ohmic contact was firstly defined. Thus, 100 nm of Ni was deposited by sputtering on the wafer backside, which was followed by rapid thermal annealing at 1050 °C for 3 min in an Ar atmosphere. An X-ray diffraction analysis evinced a nickel silicide compound with multiple diffraction peaks, which were assigned unambiguously to the Ni_2_Si phase [25]. For the Schottky contact, the two metals (Pt with WF ≅ 5.7 eV and Cr with WF ≅ 4.5 eV) with a thickness of 100 nm were deposited into the circular windows using an e-beam evaporation system. Annealing in the same conditions (600 °C for 3 min in an Ar atmosphere) was performed for the diodes from both batches, which were henceforth named *Pt/4H-SiC* and *Cr/4H-SiC*. The pad contacts and backside metallization of the final structures were achieved by evaporation of a metallic stack consisting of Ti (60 nm)/Ni (160 nm)/Au (320 nm). The final samples were diced and encapsulated in TO39 packages using Ag nano-paste for cathode bonding and Au wire-contacting for the anode connection.

The X-ray diffraction (XRD) patterns were acquired in order to evaluate the Schottky contact quality. The measurements used a 9 kW Rigaku SmartLab diffractometer (Rigaku corp., Osaka, Japan)equipped with a monochromatic CuKα1 source that provided a wavelength (λ = 0.15406 nm). During the measurements, the incidence angle of the source (ω) was fixed to 0.5°, while the detector angle (2θ) scanned from 20° to 95°. Also, the incident slit was 0.1 mm, while the receiving slits were set to 20 mm.

X-ray photoelectron spectroscopy (XPS) measurements were performed using a polychromatic Al X-ray source at 13 kV with a power of 200 W. Vacuum was maintained at ~3 × 10^−9^ mbar. The energy analyzer was a 160 mm hemispherical type with a 1D detector (ASPECT, Sigma Surface Science) (Sigma Surface Science GmbH, Taunusstein, Germany). The diameter of the analysis spot was 1.3 mm. Prior to the XPS measurements, the samples were infrared-heated to ~100 °C for 5 min and etched by Ar sputtering at 0.5 keV for 10 min. The XPS spectra were analyzed by the CasaXPS software Version 2.3. 22PR1.0 using Shirley background determination and processed to remove the satellite peaks due to the K*_β_* Al characteristic line. The binding energies were referenced to the C-C component in the C 1s signal at 284.8 eV associated with the adventitious carbon layer.

I-V characteristics of the packaged samples were measured at different temperatures between 60–500 K with a step of 20 K using a Keithley 4200 semiconductor (Keithley Corp, Cleveland, OH, USA) characterization system coupled with a Janis closed-cycle refrigerator system [14].

## 3. Results

### 3.1. Microstructural Investigations—X-ray Diffraction and X-ray Photoelectron Spectroscopy Analysis

**Figure 1:**
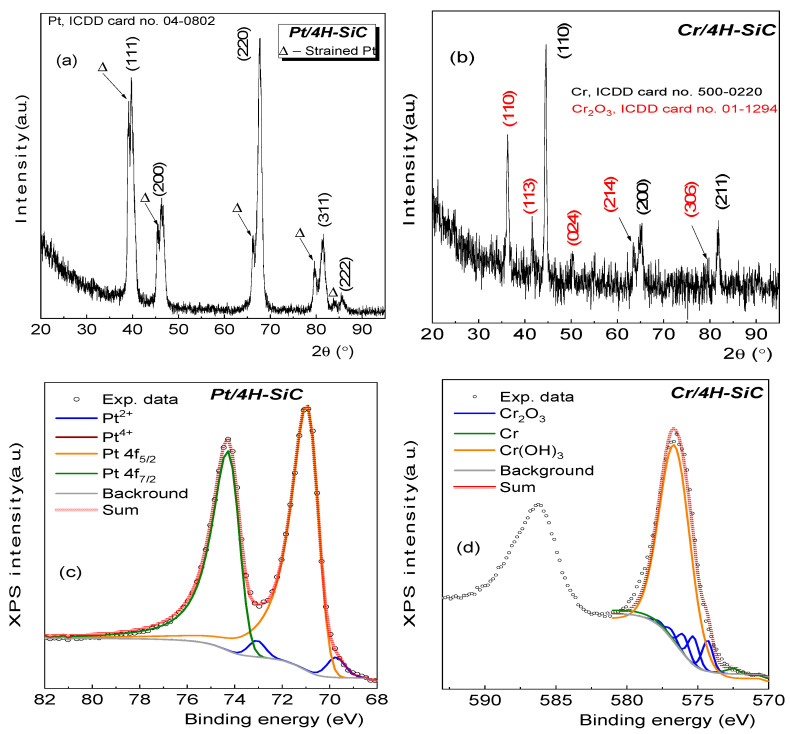
Grazing incidence XRD patterns for (**a**) *Pt/4H-SiC* and (**b**) *Cr/4H-SiC*. XPS spectra for (**c**) *Pt 4f* and (**d**) *Cr 2p* states with the corresponding fitting curves.

The interfacial reaction of Pt and Cr with SiC was studied by XRD (Figure 1a,b) and XPS (Figure 1c,d). 

Figure 1a evinces the presence of diffraction peaks at 2θ = 39.72°, 46.46°, 67.80°, 81.39°, and 85.59°. According to card no. 04-0802 of the ICDD (International Centre for Diffraction Data) database, these diffraction peaks can be attributed unambiguously to cubic Pt with a = 0.392 nm that belongs to the *Fm3m* space group. In addition, each Pt diffraction peak was accompanied at a lower 2θ = 39.17°, 45.56°, 66.34°, 79.66°, and 83.67° by a peak with a smaller intensity. These additional diffraction peaks could be assigned to strained Pt (Δ; Figure 1a) as a result of the relatively high temperature used for the sample preparation (~600 °C). According to the well-known Bragg law, lower 2θ values are associated to higher inter-planar distances. For instance, the inter-planar distances for different reflections increased as follows: from 0.226 nm to 0.230 nm (111), 0.195 nm to 0.199 nm (200), 0.138 nm to 0.141 nm (220), 0.118 nm to 0.120 nm (311), and 0.113 nm to 0.115 nm (222). Accordingly, the temperature induced the occurrence of a tensile lattice strain (ε) with different values along the atomic planes. Based on the calculated inter-planar distances, the lattice strain was: ~+1.7% (111), +2.1% (200), +2.2% (220), and +1.7% (311) and (222). The analysis also shows that no oxides or silicides were formed during the sample preparation.

In the case of *Cr/4H-SiC*, the XRD data show the presence of typical reflections of cubic Cr with a = 0.288 nm at 2θ = 44.39°, 64.58°, and 81.73°, respectively, as shown in Figure 1b. Unidentified diffraction peaks located at 2θ = 36.46°, 41.68°, 50.08°, 63.55°, and 79.40° could be assigned as (110), (113), (024), (214), and (306) reflections of Cr_2_O_3_ (ICDD, card no. 01-1294).

Further investigations related to the structure of the resulting compounds were performed by XPS for the *Pt 4f* and *Cr 2p* states. Figure 1c shows the high-resolution regions of *Pt 4f* in the range of the binding energies (BEs): 82 eV–68 eV. The BEs of these regions were calibrated using the binding energy of the adventitious carbon located at 284.8 eV.

In the fitting process, a mixed Lorentzian–Gaussian function was used to identify the compounds from the investigated sample. The presence of Pt was confirmed by the analysis of the *Pt 4f* high resolution spectra exhibiting two components, one at 71.1 eV, attributed to *4f_5/2_* (orange), and one at 74.3 eV, associated with *4f_7/2_* (olive) peaks of Pt^0^. The small asymmetry, observed in the *Pt 4f* peaks, was due to a small contribution of a peak at 69.3 eV in the *4f_5/2_* peak and a peak at 73.1 eV in the *4f_7/2_* peak associated with Pt^2+^ (blue line), which indicates that the Pt was superficially oxidized. Thus, the XPS data indicate that the Pt remained mostly in metallic form and that it did not form PtSi compounds. The results are in agreement with other studies conducted on Pt [26]. Larrieu et al. [27] investigated the evolution of *Pt 4f* with the annealing temperature, showing that the reaction of Pt to Pt_2_Si or PtSi is characterized by peaks at BEs~72.5 eV, which were absent in our case. Furthermore, XPS analysis was performed to reveal the valence state of the Cr on the SiC. Based on the fitting of the experimental data, it is revealed that the Cr^3+^ species can be further divided into oxides, which showed a discrete multiplet structure, and hydroxides, which showed only a broad peak shape at BE = 576.7 eV. Also, Biesinger et al. [28] conducted in-depth XPS studies on Cr, showing a discrete multiplet structure, whereas the hydroxide gave only a broad peak shape.

Overall, the XRD analysis showed the formation of Pt as well as of a strained Pt layer. In addition, the XPS analysis indicated a superficial oxide at the surface. No other compounds were detected. On the other hand, in the case of Cr, the XRD and XPS analyses revealed a more complex structure resulting from the thermal treatment, which included Cr hydroxide and oxide compounds. Thus, a higher degree of inhomogeneity in the sample composition was expected.

### 3.2. Electrical Characterization

#### 3.2.1. Temperature-Dependent Electrical Characteristics

**Figure 2:**
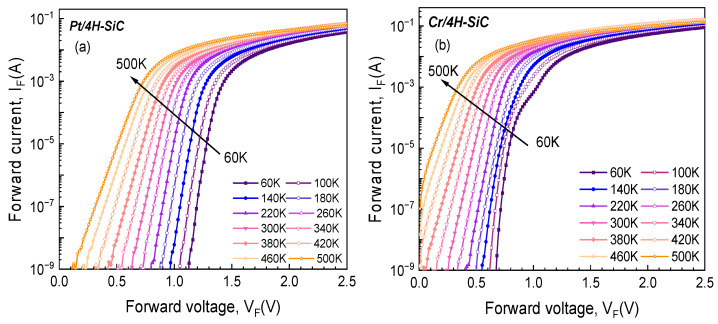
Experimental forward bias I-V characteristics of the SBDs at various temperatures: (**a**) *Pt/4H-SiC* sample; (**b**) *Cr/4H-SiC* sample.

Figure 2 shows the typical forward bias I-V characteristics of the fabricated samples in the temperature range of 60–500 K with a step of 40 K.

Exponential behavior, covering at least six orders of magnitude, was identified for each experimental sample. For the *Pt/4H-SiC* sample, this dependence was evinced even at 500 K, while, for *Cr/4H-SiC*, the lower WF (and, consequently, lower SBH) led to a much higher increase in the saturation current with temperature.

#### 3.2.2. Standard SBD Characterization 

The I-V-T characteristics of an ideal SBD are governed by the thermionic emission (TE) law [29]:

(1) $$I_{F}≅I_{S}exp\left(\frac{V_{F}-I_{F}R_{S}}{{nV}_{th}}\right),$$

where $R_{S}$ is the series resistance, *n* is the ideality factor, $V_{th}$ = kT/q is the thermal voltage, and $I_{S}$ is the saturation current,

(2) $$I_{S}≅A_{n}A_{S}T^{2}exp\left(-\frac{\Phi_{Bn,T}}{V_{th}}\right),$$

where $A_{S}$ is the designed contact area, $A_{n}$ is the Richardson constant for electrons (146 A/K^2^ cm^2^ for n-type 4H-SiC), and $\Phi_{Bn,T}$ is the conventional Schottky barrier height. 

**Figure 3:**
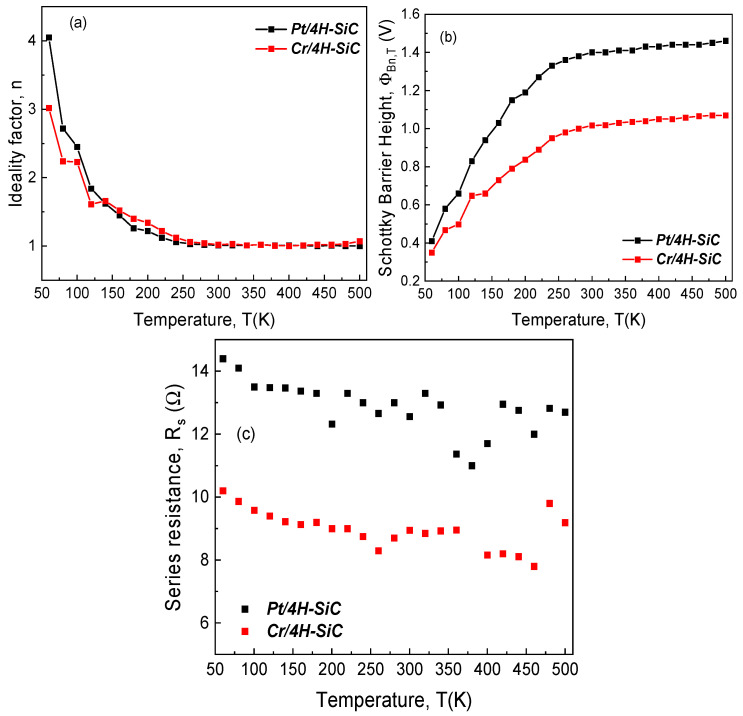
Temperature dependence of electrical parameters for the fabricated SiC SBD samples with Pt and Cr: (**a**) ideality factor; (**b**) Schottky barrier height; (**c**) series resistance.

The standard technique for extracting the main electrical parameters of an SBD entails the representation of ln ($I_{F}$) as a function of $V_{F}$, followed by linear fitting. The ideality factor and SBH are determined from the slope and intercept of this fit. For series resistance ($R_{S}$) determinations, a linear fit of the I-V plot in the high voltage domain is carried out. These electrical parameters, for the two fabricated samples, were extracted from the data in Figure 2. Their variation with temperature was plotted and is shown in Figure 3. According to the TE theory, an ideal Schottky contact yields a temperature-stable, constant SBH and ideality factor.

Additionally, for SBDs with reasonably uniform contacts, the ideality factor should exhibit values close to unity. In our case, this situation corresponded to both the *Pt/4H-SiC* and *Cr/4H-SiC* samples only in the 260–500 K temperature interval. This range was also associated with a near-constant value for the Schottky barrier height. Conversely, a significant temperature dependence for these parameters was observed in the 60–240 K range. The behavior, coupled with the XRD findings, confirm that the devices’ contacts were inhomogeneous.

#### 3.2.3. State-of-the-Art Contact-Inhomogeneity Analysis

**Figure 4:**
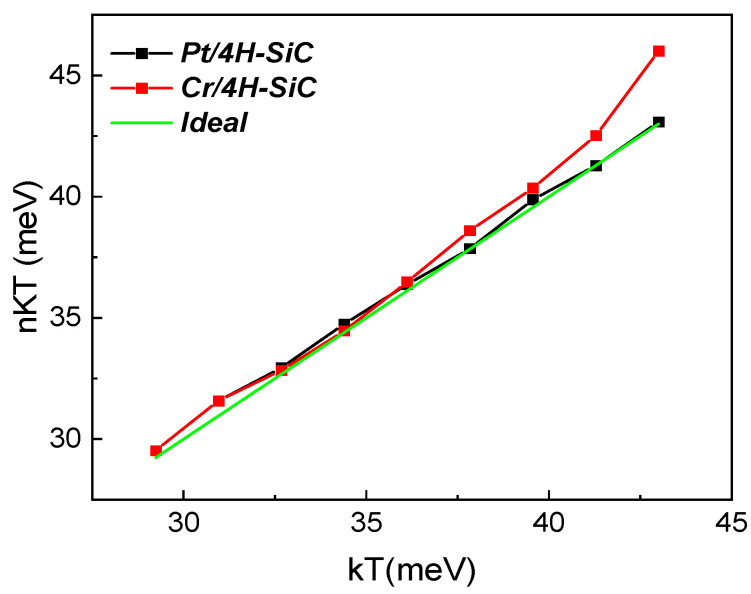
Plot of nkT vs. kT for both the fabricated samples. The ideal behavior (*n* = 1) is also reported as reference (green line).

Multiple techniques were carried out in order to comprehensively evince the degree of contact inhomogeneity for the investigated diodes. Firstly, we evaluated the deviation from the ideal behavior, which is illustrated by the nkT vs. kT plot depicted in Figure 4. For this representation, the 340–500 K temperature range was considered, corresponding to an interval where the ideality factor was nearly constant.

Slight deviations from the ideal case (*n* = 1; green line in Figure 4) were observed. This anomaly is normally attributed to Schottky barrier non-uniformity [30]. In such cases, the ideality factor temperature dependence can be expressed as [31]:

(3) $$n=1+\frac{T_{0}}{T},$$

where *T*_0_ ≠ 0 is referred to as a *T*_0_ anomaly [21]. A high value for *T*_0_ corresponds to a higher degree of inhomogeneity. Values of 14.6 K for the *Pt/4H-SiC* sample and 50.1 K for the *Cr/4H-SiC* one were obtained from (3) after linear fitting was conducted on the curves illustrated in Figure 4. The relatively high value corresponding to the *Cr/4H-SiC* diode occurred mostly because of the data point at 500 K, indicating that this temperature level was beyond the normal capabilities for this type of contact. Excluding it from the analysis yielded *T*_0_ = 20.8 K for *Cr/4H-SiC*, which was much more in agreement with its Pt counterpart. While performing this rudimentary *T*_0_ anomaly technique can serve as a quick way to confirm that contact inhomogeneities do influence electrical characteristics significantly, it does not offer any physically relevant parameters.

A more thorough characterization can be performed using models that consider the well-known parallel conduction theory [21]. According to this, an experimental Schottky contact has numerous low-area regions (“patches”) with independent barrier heights. If a Gaussian distribution of these patches is considered at the interface [32] with a mean Schottky carrier height ($\Phi_{Bn}^{0}$) and standard deviation (σ), the conventional SBH temperature dependence (Figure 3b) can be expressed according to the following equation [32]:

(4) $$\Phi_{Bn,T}=\Phi_{Bn}^{0}-\frac{q\sigma^{2}}{2kT}.$$

**Figure 5:**
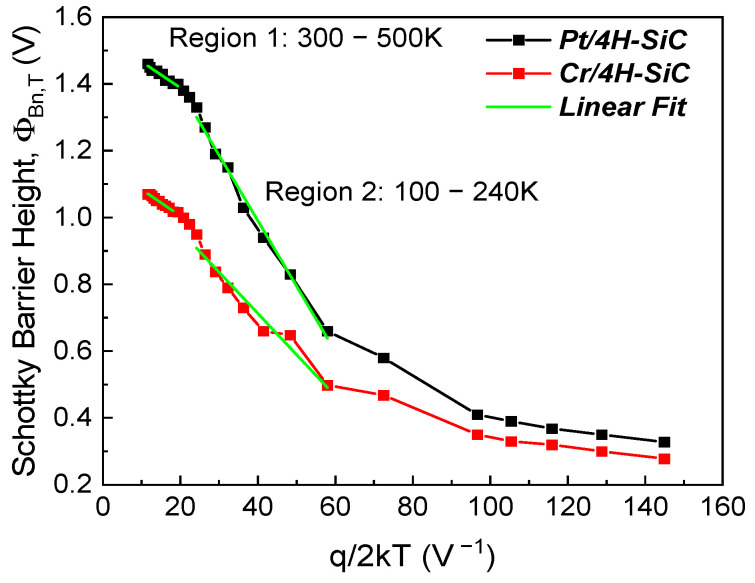
Conventional SBH vs. q/2kT.

Representing $\Phi_{Bn,T}$ as a function of q/2kT (Figure 5), we can determine both $\Phi_{Bn}^{0}$ and *σ* from the resulting intercept and slope.

Two linear regions can be identified on the graph in Figure 5, demonstrating that at least two Gaussian distributions were found on the contact’s surface [33]. The extracted mean SBH and its standard deviation for the two temperature ranges are shown in Table 1 for both samples. The mean SBH for the *Pt/4H-SiC* sample was higher than that of the *Cr/4H-SiC* sample over both temperature intervals due to the difference in the metal WF values for the Schottky metals.

Note that a third region can theoretically be identified for the 60–100 K range. At such low temperature levels, however, conventionally extracted SBH values are significantly affected by errors stemming from the bias interval window of analysis and, possibly, carrier freeze-out [34,35]. Since the Gaussian distribution model does not consider such effects, no significance can be derived from applying the technique to the 60–100 K measurements.

The discrepancies between the values determined using this technique and their counterparts from the conventional method (see Figure 3b) make this analysis unable to explain the overall behavior of our fabricated samples. As we can see in Table 1, the mean SBH presented higher values over both temperature intervals than what the conventional SBH trend would suggest (see Figure 3b). Practically, this means that the determined $\Phi_{Bn}^{0}$ was not the dominant one.

Since the behavior of the SBD samples was closer to the ideal (*n* < 1.07) over 300–500 K, this higher temperature interval will be referenced further in our analysis of the contact inhomogeneity.

Additional information about the impact of the contact inhomogeneity is given by the Richardson plot [31], from which both an effective SBH $\left(\Phi_{Bn-eff}\right)$ and active area (*A*_S-eff_) can be determined. The Richardson plots for our samples are governed by the expression:

(5) $$ln\left(\frac{I_{F}}{T^{2}}\right)=ln\left(A_{S}A^{*}\right)-\frac{q{(\Phi }_{Bn-eff}-\frac{V_{F}}{n_{med}})}{kT},$$

where $n_{med}$ represents the mean ideality factor value over a certain temperature interval. 

**Figure 6:**
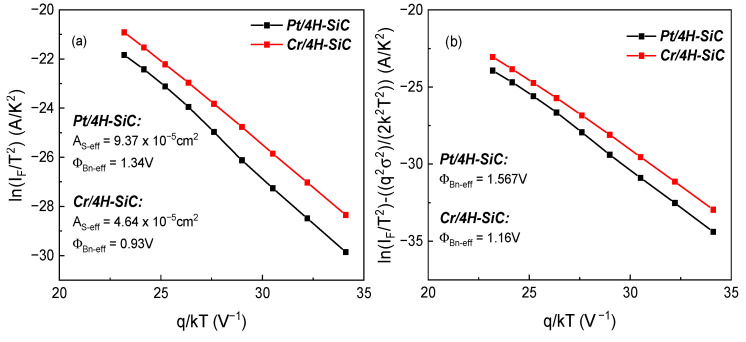
(**a**) Optimal Richardson plot; (**b**) optimal modified Richardson plot, using standard deviation from Table 1 (300–500 K).

Using the standard deviation values determined before (see Table 1), we can construct a modified Richardson plot,

(6) $$ln\left(\frac{I_{F}}{T^{2}}\right)-\left(\frac{q^{2}\sigma^{2}}{2k^{2}T^{2}}\right)=ln\left(A_{S}A^{*}\right)-\frac{q{(\Phi }_{Bn-eff}-\frac{V_{F}}{n_{med}})}{kT},$$

where σ is the standard deviation determined using the conventional SBH vs. q/2kT plot from Figure 5 with the values in Table 1 for both temperature intervals: low (100–240 K) and high (300–500 K). Since $n_{med}$ is truly representative for measurements only in the high-temperature region, we constructed Richardson plots by taking several bias voltages from the characteristics only in this domain, which was further restricted to 340–500 K. This approach was taken in order to ensure that all the selected curves exhibited exponential behavior for each *V_F_*. The voltage interval of 0.5–0.75 V was identified for *Pt/4H-SiC* and 0.2–0.4 V for *Cr/4H-SiC*. The effective SBH was determined from these intervals, and the optimum voltage for constructing both the Richardson and modified Richardson plots was selected such that fitting errors were minimized. Thus, a bias voltage of 0.6 V was chosen for the *Pt/4H-SiC* sample and 0.25 V for *Cr/4H-SiC*. The plots are given in Figure 6. 

The effective active area ($A_{S-eff}$) of the samples was determined as being one order of magnitude smaller than the designed one (~12.56 × 10^−4^ cm^2^). This is another probative indicator of the occurrence of inhomogeneity on the contact surface. Moreover, the *Cr/4H-SiC* sample exhibited an $A_{S-eff}$ two times lower than *Pt/4H-SiC*, indicating a higher degree of inhomogeneity. Accordingly, the current flow through the device is favored by the small, low-barrier patches located on the contact surface. The effective SBH value, determined using the standard Richardson plot, is also given in Figure 6. It better corresponded to the electrical behavior of the fabricated samples, as it was in suitable agreement with the variation in the trend of the conventional SBH values plotted in Figure 3b. Conversely, the SBH value obtained from the modified Richardson plot naturally mimicked the one determined using the Werner and Güttler plot (see Figure 5). It is, theoretically, the extrapolation at infinite temperature of the governing barrier height. Hence, in practice, this Gaussian distribution method does not produce relevant parameters for highly inhomogeneous devices. The temperature threshold after which these extracted barrier heights would become indicative of actual device current flow far exceeds operational levels.

**Figure 7:**
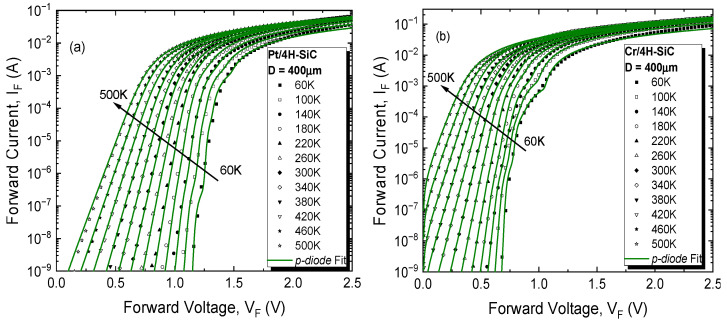
*p-diode* model-fitted curves for 4H-SiC Schottky diode samples with (**a**) Pt and (**b**) Cr metals.

Finally, for a complete elucidation of the fabricated samples’ electrical behavior over the entire investigated domain, a more comprehensive approach was undertaken. It was based on our developed *p-diode* model [24], which also uses the parallel conduction theory as the underlying principle. According to it, a real Schottky contact behaves like a grouping of multiple parallel-connected near-ideal diodes (*n* capped at 1.03), each with its specific barrier height and non-uniformity parameter (*p_eff_*, giving a quantitative depiction of the occupied area proportion). Distinctively from the Gaussian approach, each parallel diode is also associated with a series resistance that can limit its current contribution to the overall I_F_ as the bias increases. The forward curves of both the *Pt/4H-SiC* and *Cr/4H-SiC* samples were characterized with the *p-diode* technique over the entire 60–500 K range. The model-fitted curves are depicted in Figure 7.

**Figure 8:**
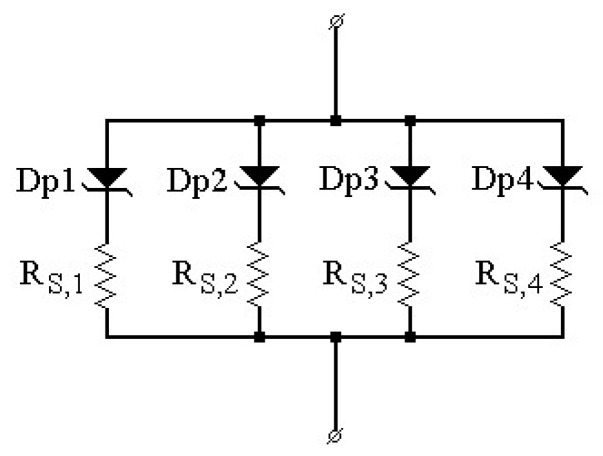
*p-diode* model equivalent schematic for 4H-SiC Schottky diode samples with Pt and Cr metals.

An excellent agreement between the fitted curves and experimental measurements can be observed, even at low temperatures, where contact inhomogeneity determines strong deviations from the exponential I_F_–V_F_ dependence. Modeling at such near-cryogenic levels is possible distinctly because of the *p-diode*’s consideration of patch resistive effects (ignored in the Gaussian distribution model). Four parallel diodes (Dp1–Dp4, Figure 8) were necessary to fully replicate the electrical behavior for both samples in the encompassing temperature span, corresponding to different barrier zones. The obtained parameters are given in Table 2. These regions were associated with the different compounds on the contact surface evinced by the XRD and XPS analyses.

For the *Pt/4H-SiC* sample, Pt-Dp1, covering most of the overall contact surface, exhibited the highest barrier height and, at low temperatures, was only influential at a high bias. As the temperature increased, this parallel diode began to contribute significant current over the entire *V*_F_ range. Conversely, Pt-Dp4 was only prominent in the low-temperature, low-bias regions, with its impact becoming negligible at higher *T* and *V*_F_ levels. Parallel diodes Pt-Dp2 and Pt-Dp3 had comparable influence on the forward characteristics throughout the entire bias and temperature intervals. Their lumped contributions were especially relevant in the 300–500 K domain, as also confirmed by the $\Phi_{Bn}$ values similar to that obtained from the Richardson plot (see Figure 6). 

For the *Cr/4H-SiC* sample, Cr-Dp2 was the main current contributor, which was once again verified by the results obtained from the Richardson plots. Cr-Dp3 and Cr-Dp4 were responsible for the current flow in the lower ranges of bias and temperature, while Cr-Dp1 mostly influenced *I*_F_ at the top end of the temperatures at high *V*_F_.

The *p-diode* analysis completely explains the forward electrical behavior of the investigated samples throughout the entire temperature domain. Both exhibited a considerable degree of contact inhomogeneity, with multiple current paths becoming preferential as the bias and thermal conditions evolved. Even so, suitable I_F_ levels can be found, where the samples essentially behaved like ideal Schottky diodes, enabling their use in temperature and gas sensing applications. For such uses, the Pt samples are more desirable due to their overall larger barrier height in order to obtain both a higher sensitivity and a wider operable temperature range [36].

An important conclusion of the comprehensive inhomogeneity analysis is that all of the employed techniques, apart from the *p-diode* method, either analyzed the Schottky contact area as a whole or required restricted temperature intervals in order to produce trustworthy results. While being useful tools for preliminary contact quality diagnosis, they must be accompanied by *p-diode* modeling in order to accurately and completely assess the forward electrical behavior of such wide-temperature-range SiC diodes.

## 4. Conclusions

This paper analyzed the contact inhomogeneity of Pt/SiC and Cr/SiC. The fabricated samples were subjected to XRD and XPS analyses, revealing possible inhomogeneity sources. For the *Pt/4H-SiC* sample, slight traces of oxides and a strained layer were identified. In the case of *Cr/4H-SiC*, more pronounced inhomogeneity was evinced, stemming from hydroxide and oxide compounds. The conventional electrical characterization demonstrated important variations in the barrier height and ideality factor with temperature, which confirmed the formation of a non-uniform contact. Subsequent inhomogeneity modeling employed techniques of gradually increasing complexity, which confirmed that the *Cr/4H-SiC* diode was more affected by this spurious influence.

The forward characteristics of both samples were completely modeled with our *p-diode* technique over the entire investigated domain. Each of the samples behaved essentially as four parallel-connected ideal diodes, with variable influence on the current conduction, depending on the temperature and bias levels. The *Pt/4H-SiC* diode’s current was mainly given by a contact region with a barrier of 1.3–1.35 V. For the *Cr/4H-SiC* sample, a main barrier of 0.93 V governed the current conduction. Both of these results were corroborated by the ones obtained from the Richardson plots and conventional SBH extraction in the plateaus corresponding to the high-temperature domain.

Employing the *p-diode* modeling was crucial in order to identify the suitable operable bias and temperature conditions for these samples.

## Figures and Tables

**Table 1:** Extracted values for the mean SBH and standard deviation for different temperature intervals.

Extracted Parameters				
Temperature Range, *T* (K)	*Pt/4H-SiC* Sample		*Cr/4H-SiC* Sample	
	$\Phi_{Bn}^{0}$(V)	*σ* (V)	$\Phi_{Bn}^{0}$(V)	*σ* (V)
100–240	1.774	0.134	1.206	0.111
300–500	1.544	0.088	1.163	0.089

**Table 2:** Extracted *p-diode* model parameters.

Sample	Parallel Diodes	*Φ*_Bn_ [V]	*n*	*p_eff_*	*R*_S_ [Ω]
*Pt/4H-SiC*	Pt-Dp1	1.51	1.03	0.51	44–60
	Pt-Dp2	1.35		3.04	150–48
	Pt-Dp3	1.3		4.39	150–800
	Pt-Dp4	1.21		9.26	200 k–40 k
*Cr/4H-SiC*	Cr-Dp1	1.1		0.37	14.2–21.9
	Cr-Dp2	0.93		3.73	50–430
	Cr-Dp3	0.8		10.98	300–550
	Cr-Dp4	0.73		13.39	~15 k

## Data Availability

Data are contained within the article.
